# Homœpathy and Allopathy

**Published:** 1884-04

**Authors:** C. S. Carr


### HOMŒPATHY AND ALLOPATHY.

C. S. CARR, M. D.

Under the above titles are marshalled the two greatest factions of the medical art. The claims of one is supposed to totally repudiate that of the other, and the only point at which agreement is even approximated is when promptly classing all persons fools who, having patiently listened to both claims, are convinced of the truth of neither, and in consequence seek some other method of cure when sick.

According to Allopathy, Homœpathy is a fraud. According to Homœpathy, Allopathy is ditto. Listening to both claims, one is compelled to reason something like this:

Since the two theories dispute each other, one only *can* be right. One *must* be wrong, both *may* be. If one is wrong and the other right, then, it of necessity follows that there would be a great difference in the results of their application; and statistics would soon tell the story. But if it be that *both* are wrong

(which is the only other hypothesis), then would it follow that statistics would not decide anything, and the present paradoxical condition of the controversy ensue, the whole matter resting on the unsupported claims of each.

We are not aware that any positive statistical evidence has been obtained in favor or against the claims of either, which is exactly as it should be, if both were wrong. Among all the hypotheses projected in reference to the art of medication,one possible hypothesis (just as legitimate as any other) seems to have been entirely overlooked, viz: That all medication is entirely impotent to mitigate disease; and, at best, can only modify or complicate the derangements set up by disease. If this be the true hypothesis, then, that which is the darkest mystery with any other possible hypothesis, becomes as clear as the mid-day sun. If all systems of medication are equally impotent, then *cœteris paribus* would they all be equally successful. So far as statistics permit us to judge they are, as systems, equally successful and notably equally unsuccessful, which fact is wholly incompatible with the hypothesis that one is wrong and the other right. Is it possible that two "doctors" living in the same locality, practicing among the same people, one practicing a system of medication that contradicts every item of the practice that the other adheres to, can be equally successful if either are right? Is it possible that one can *destroy* life and the other *save* it, and yet no differences appear in their mortal statistics?

Not only does the mistaken one do no good, but if he does anything he must do harm. Now, if they are equally false, then, although they differ, they would give us about the same results. We do not forget that there are many other systems of medicine equally as successful in results if not in number of adherents, who agree in only one thing, viz: that both the Allopathic and the Homœpathic are false systems,which claim, if granted, will also prove their own systems false, unless they are able to show a decided improvement in results over those of the alledged false systems. What are the real issues that constitute the different practices called Homœpathic and Allopathic?

All remedial agents may be divided into four classes, Food, Antidotes, Palliatives and Specifics.

By food is meant all things sold by druggists which purport to contain nutrition, or in any way furnish the human system with substance which, when taken into the stomach, can be appropriated as nutrition for the purposes of force-making, or tissue-building.

We are taught (save in medical works) that no substance can be appropriated to uses of the animal economy, which has not first been appropriated by a vegetable; but, Allopathic medical works teach us that to make blood we have only to give crude iron; to make nerve tissue. crude phosphorus.

The Allopaths are the champions of this class of drugs, the Homœpaths, if true to the teaching of their school, repudiate them all, and seek to remove the cause of the mal-nutrition by "specific" treatment.

Hence, all the precious blood making compounds of the Allopath are rubbish to the Homœpath. Before us, at this moment, are two medical works, one a leading Allopathic, the the other a leading Homœpathic work. We consult them upon such subjects as Anæmia, Chlorosis, etc., and what do we find? Not a single point of agreement or similarity between their teachlngs! Not one!

And yet, who will undertake to say one school is not as successful as the other, in the treatment of such conditions?

Antidotes include all substances which, when taken, have the effect of rendering inert or modifying the action of poisons already taken. Toxicology is the science of antidotes, and since it has to deal with undoubted facts there *is*, nor never *has been*, any dispute on this subject. Toxicology, like physiology, surgery, and anatomy, is not peculiar to any school of medicine, but is as much the property of one as the other. It is only where knowledge is impossible and speculation rife that schools begin. The least known about a subject, the more dogmatic and arrogant becomes its partisans!

It is a notorious fact that success in the practice of medicine does in no way depend upon a knowledge of the subject, but upon other qualifications of the practitioner, quite separate from medical lore. Palliatives are medicines that, while they possess no real curative virtues, yet they are able to remove or modify some prominent symptom, as pain, constipation, sleeplessness, etc., etc. This class of remedies constitute about all the drugs found in an ordinary drug store, and without exception are never used by the genuine Homœpath. While the Allopath uses remedies which are simply palliative, the Homœpath professedly *never* uses them. While the Homœpath professedly uses nothing but specific remedies, the Allopath denies the existence of any such drug.

The Allopath uses palliatives simply because he does not believe in the existence of a drug that has any specific curative properties. The Homœpath believes he has a specific remedy for every possible ill that human flesh is heir to, and, consequently, never uses palliatives, unless, indeed, he departs from the teachings of his school. The Allopath believes the Homœpath does *nothing;* the Homœpath believes the Allopath does *harm!* And yet, they practice side by side, year after year, and so far as results go, there is no difference in their success! Some Allopaths are successful in making a reputation and a fortune, and some are not. Some Homœpaths are equally successful, and some are not. It all depends on the *man*, and *not* to the school of medicine to which he belongs!

The "Alterative" is the Allopath's specific, if he has any, but the Homœpath disbelieves both its application and mode of administration. The alterative is not alterative to the same condition that the specific is specific!

One conscientiously avoids that which the other is careful to do; the other never could be persuaded to do, on any account, the things upon which the other supposes his success depends. And yet, both are equally successful. If one is right and the other wrong, the paradox is complete. If both are wrong, then, what seems a paradox, follows as a matter of course. A specific is a remedy which is supposed to bear a specific relation to the *cause* of disease which it removes, without producing any symptom save the cure of the disease. The Homœpath religiously believes that there is in nature a specific remedy for every possible abnormal condition of the human system, and, further, claims he is in the possession of at least four hundred such specifics. With very few exceptions, the Allopath does not believe that a specific for disease has ever been discovered. He simply scoffs at the idea of specific treatment, and continues the use of his palliatives. The Homœpath, as doggedly, continues to use his specifics, and claims never to use palliatives. They never prescribe the same drug with the same intent or application, during their whole lives, and yet both acquire great reputations in the cure of disease.

"Mercury, in any of its forms, possesses no curative virtue, in mucus inflammations," says the Allopath. "It is a specific for mucus inflammations," says the Homœpath. While they occasionally use the same drugs, they *never* use them with the same intent or application. Two persons of the same age and physical condition are taken sick with typhoid fever. Their houses adjoin. One employs a Homœpath, the other an Allopath. Both take the medicine given them. Not a single drug is used by the Allopath that the Homœpath uses, and *vice versa.* The drugs, doses, and theory of treatment, are entirely different. The medical treatment does not agree in any *single detail* or particular! One supposes he must interrupt his patient's temperature, or his patient will die; the other quietly ignores the temperature, and silently gives his specifics. One supposes that the recovery of his patient depends upon his prompt and persistent use of quinine; the other would not give his patient a single dose of it, as he values his recovery, and relies on his baptisia, etc.

If both recover, each doctor gets the glory of curing his patient. If both die, a shadow of doubt never crosses the mind of the surviving friends that anything was left undone to save the life of the lost one.

Both gain their reputation in the treatment of those diseases that tend by their own peculiarities to recover. All organic diseases are totally incurable by any or all systems of medication, and the reason is, that they have no inherent tendency towards spontaneous recovery. *Only such diseases are curable as tend to cure themselves!* Every second person on earth has some little, annoying, perhaps insignificant, but *incurable* derangement. Thousands upon thousands of poor deluded mortals afflicted with some slight derangement depending on some bad habit, are going from doctor to doctor in vain, to find pardon for their physical transgressions, until, disgusted, they quit, with the same disease they began with, plus a ruined stomach, and deplete exchequer. The *modus operandi* of any drug is not positively known; the knowledge of the operation of drugs is wholly empirical. No one could even *guess* how a drug would operate until it had been tried, and even after its effect has been observed, *why* it operated as it did, must forever remain a *guess*, without a single data to rest upon. The chemical analysis of a drug throws no light upon its peculiar effect upon the human body. The chemical analyses of some of the most deadly poisons are almost identical with the most nutritious substances known, and why one is poison and the other nutritious, is a mystery no one can explain. Experience alone can teach us *how* drugs operate. Therapeutics can *only* speculate as to *why* they operated so. No light whatever can be had as to *how* a drug will operate until after it has been tried. As to the real essence of disease, not even a conjecture has ever disturbed the stillness of the darkness that envelops the whole subject. Life, vitality, animal force, animal heat, are but little understood; how could it be expected that disturbances of either or each of these would be ?

If the normal manifestations of life are not understood, how can we expect to understand the abnormal manifestations of life, or "disease," as it is called ? If we do not know *why* and *how* a watch runs at all, how can we be supposed to know why it does *not* run right ? Would we trust a person to regulate a watch for us who was in doubt as to whether the motion of the wheels was inherent in the wheels, or resided in some secret, miraculous corner of the watch ? If we are not clear as to the utility, origin, or rationale of the temperature of the human body, how can we be expected to intelligently regulate its abnormal deviations ? The question is not whether we can affect the temperature or not (for we know we can), but the question is whether it is done with sufficient intelligence as to the result of our interference. No system of quackery, however fallacious, is without its testimonials; genuine, heartfelt testimonials, too! For, having been sick, they are well again, and, like all the rest of us, they have assumed that the treatment was the *cause* of the recovery.

What is to be the future developments of pathological research we do not predict. What the microscope may reveal to aid the physician in the future, we can not say; but up to date, not one single step has been made toward rescuing the art of medication from *absolute empiracism.* The pathology, etiology, of any disease is not sufficiently understood to suggest anything that will aid the therapeutician; the therapeutician does not understand, with sufficient clearness, the *modus operandi* of any drug to be of any use to the pathologist. As yet, the two have never been made to articulate; only enough has been revealed to show the student of medicine what a yawning abyss, of the darkest ignorance, continually divides the two. Pathology only, as yet, has been able to furnish objective material, to gratify the harmless inquisitiveness of the medical student, while therapeutics has only been able to furnish its quota of subjective material, to keep alive a ceaseless bombardment of speculations, couched in that vaguest, say-much-and-mean-little language known only to the medical meta-physician. *Amicus Plato, amicus Socrates, sed majis amica veritas.*

